# New Appliances and Things Medical

**Published:** 1898-01-01

**Authors:** 


### NEW APPLIANCES AND THINGS MEDICAL.

[We shall be glad to receive, at our Office, 28 & 29, Southampton Street. Strand, London, W.C., from the manufacturers, specimens of all new preparations and appliances which may be brought out from time to time.]

### CHAMPAGNE SANS ALCOHOL.

(FIRST SWISS WINE SANS ALCOHOL COMPANY, LIMITED, BERNE; AND 39, EASTCHEAP, E.C.)

The unfermented and non-alcoholic wines of the First Swiss Wine Sans Alcohol Company are guaranteed to be prepared from fresh grapes, and by a process of sterilisation, so that fermentation changes are avoided, the juice of the grape is obtained in the form of a beverage without alcohol. The wines thus prepared are said to retain their flavour and properties for years. The sample of "Champagne Sans Alcohol" submitted to us bears out on analysis the characters claimed for these wines. It contained no alcohol. It had a specific gravity of 1·067, contained 16·5 per cent. extractives, 0·3 per cent. mineral constituents, and 0·9 per cent. free acid, calculated as tartaric acid. No added preservative could be detected. The wine was well aërated, nd had a characteristic vinous flavour.

